# Interferon-alpha/beta receptor deficiency enhances susceptibility to Powassan virus infection in mice

**DOI:** 10.3389/fmicb.2025.1678861

**Published:** 2025-10-07

**Authors:** Amany Elsharkawy, Heather Pathak, Chinonye Dim, Mukesh Kumar

**Affiliations:** ^1^Department of Biology, College of Arts and Sciences, Georgia State University, Atlanta, GA, United States; ^2^Center of Diagnostics and Therapeutics, Georgia State University, Atlanta, GA, United States

**Keywords:** orthoflavivirus, Powassan virus, inflammation, interferon response, interferon-alpha/beta receptor

## Abstract

Powassan virus (POWV) is a tick-borne flavivirus that causes neurotropic disease in humans. POWV causes fatal encephalitis and meningitis in 10% of human cases and long-term neurological sequelae in 50% of surviving patients. While innate antiviral responses have been extensively studied in mosquito-borne flavivirus infections, they remain less well characterized in the context of tick-borne flaviviruses. In this study, we investigated the role of interferon α/β receptor in the pathogenesis of POWV infection *in vivo*. Herein, we showed that unlike wild-type (WT) mice, interferon α/β receptor-deficient (*Ifnar^−/−^*) mice were highly susceptible to POWV and rapidly succumbed to infection. Low inoculum dosage resulted in 100% mortality rate in *Ifnar^−/−^* mice early after infection. Higher levels of viremia accompanied by increased serum levels of proinflammatory cytokines and chemokines were observed in *Ifnar^−/−^* mice. Further, we detected significantly higher virus levels in the peripheral tissues including spleen, liver and kidney in *Ifnar^−/−^* mice compared to WT mice. Subsequent analyses revealed marked pathology and elevated inflammatory responses in the peripheral organs of *Ifnar^−/−^* mice. Additionally, *Ifnar^−/−^* mice showed a stunted immune response in the spleen with significantly decreased numbers of B cells, monocytes, and neutrophils. While WT mice exhibited increased splenic accumulation of Ly6C^+^ cells, this recruitment was markedly impaired in *Ifnar^−/−^* mice. Notably, viral load quantification and immunofluorescence analysis showed no significant difference in brain viral load between WT and *Ifnar^−/−^* mice; however, *Ifnar^−/−^* mice displayed elevated inflammatory response in the brain. These data suggest that the rapid mortality observed in *Ifnar^−/−^* mice is due to uncontrolled virus dissemination and excessive inflammation in the periphery rather than brain infection. Collectively, our data reveal that the type-I interferon response restricts viral tropism and pathogenesis of POWV in mice.

## 1 Introduction

Powassan virus (POWV) is an emerging orthoflavivirus in North America that causes severe neuroinvasive disease characterized by encephalitis, meningitis, and encephalomeningitis (Hermance and Thangamani, 2017). POWV is a positive sense single-stranded RNA (+ssRNA) virus with approximately 11 kb genome. POWV is vectored by several *Ixodes* tick species including *Ixodes scapularis* and *Ixodes cookei*. POWV is amplified in multiple animal host reservoirs including groundhogs, squirrels, skunks, opossum, chipmunks, and potentially some bird species (Hassett and Thangamani, 2021). Humans are dead-end hosts, and human infection is often considered as a spill-over event. Since its discovery in Powassan, Ontario, POWV continues to spread through Canada and the Northeastern United States (McLEAN and Donohue, 1959). The clinical manifestations for POWV infection can range from mild flu-like symptoms to advanced neurological involvement. Neurological symptoms can consist of headache, ataxia, aphasia, blurry vision, seizures, and acute flaccid paralysis. Case fatality rates for patients that progress onto neurological disease is 10–30 percent. And 50 percent of surviving patients experience long-term neurological sequalae (Campbell and Krause, 2020; Fatmi et al., 2017). Currently, there are no treatments or vaccines available against POWV.

Upon viral infection, pattern recognition receptors (PRRs) induce the signaling of the interferon (IFN) response to mediate antiviral activity (O’Neill and Bowie, 2010). Unlike the restricted expression of type-II (IFNγ) and type-III IFN (IFNλ) receptors, type-I IFN (IFNα/β) receptors are ubiquitously expressed on all nucleated cells (Trinchieri, 2010). Signaling through IFN receptors induces an antiviral state through the expression of IFN-stimulated genes (ISGs), interferon regulatory transcription factors (IRF), IFN-stimulated response elements (ISREs), nuclear factor kappa B (NF-ΚB) and the JAK/STAT pathways (McNab et al., 2015). A robust and early IFN response is critical for controlling viral replication. Therefore, most RNA viruses, including flaviviruses, have developed various evasion strategies to circumvent the host IFN response (García-Sastre, 2017; Lubick et al., 2015; McNab et al., 2015).

Type-I IFN signaling is a critical component of antiviral innate immunity and contributes significantly to virus pathogenicity. For example, type-I interferon response protects mice from fatal neurotropic disease following infection with Langat virus (LGTV) and pseudorabies virus (Weber et al., 2014; Wei et al., 2017). Treatment with anti-IFN receptor monoclonal antibody significantly enhanced the susceptibility of mice to POWV infection (VanBlargan et al., 2018). In West Nile virus (WNV) infection, resistance to type-I interferon response is a determinant of viral replication and virulence *in vitro* and *in vivo* (Keller et al., 2006). Additionally, IFNα/β receptor plays an important role in resisting WNV and enhancing neuronal survival (Samuel and Diamond, 2005).

The role of the IFN-α/β receptor in the pathogenesis of WNV, ZIKA and tick-borne flaviviruses has been investigated (Lazear et al., 2016; Samuel and Diamond, 2005). However, its specific contribution to POWV pathogenesis remains poorly understood. Type-I interferon receptors knockout mice (*Ifnar^−/−^*) have been previously used to elucidate the contribution of type I interferon responses to host defense and disease outcome (Chotiwan et al., 2023; Lazear et al., 2016; Li et al., 2017). In the present study, we evaluated the role of IFNα/β receptor in controlling POWV replication, spread, and tissue tropism. We infected wild-type (WT) mice and *Ifnar^−/−^* subcutaneously with POWV LB strain. We determined virus burden and assessed POWV-induced pathology in various organs. We also evaluated cellular response in the spleen via flow cytometric analyses. Additionally, we assessed local and systemic inflammation through cytokine and chemokine level measurements.

## 2 Materials and methods

### 2.1 Ethics statement

The protocol was approved by the GSU Institutional Animal Care and Use Committee (Protocol number A24041). All animal experiments were conducted in the certified animal biosafety level 3 (ABSL-3) facility in consultation with veterinary and animal care staff at Georgia State University (GSU).

### 2.2 POWV infection in mice

POWV, LB strain (BEI resources, NR-51181), was used for all the experiments. Virus was propagated once in BHK-21 cells. C57BL/6 mice were purchased from The Jackson Laboratory and bred in the vivarium at GSU. *Ifnar^−/−^* mice, which are deficient in the IFNα/β receptor (C57BL/6 background), were obtained from the Mutant Mouse Resource and Research Center (MMRRC strain no. 032045-JAX). Equal numbers of males and females were used for each experiment. Six-week-old C57BL/6 and *Ifnar^−/−^* mice were inoculated with 20 μL of 10 or 100 plaque forming units (PFU) POWV or PBS (mock) via the footpad route. Infected mice were monitored, and weights were documented for each group. At euthanasia, animals were anesthetized with isoflurane and perfused with cold 1X PBS. Tissues were harvested, and flash-frozen in 2-methylbutane or preserved in 4% paraformaldehyde.

### 2.3 Viral burden quantification

Tissues were pounded and lysed in RLT buffer. Total RNA was extracted using a Qiagen RNeasy Mini kit (Qiagen, Germantown, MD, United States) (Elsharkawy et al., 2025b). cDNA library was created using iScript™ cDNA synthesis kit (Biorad, Cat#1708891). The cDNA was used for qPCR using SsoAdvanced™ Universal Probes Supermix (Biorad, Cat# 1725281) (Auroni et al., 2023; Jahantigh et al., 2025). Viral RNA levels were measured with primers and probes specific for POWV (Table 1). Viral genome copies were calculated using a standard curve and expressed per μg of total RNA. The viral load in the serum was measured by plaque formation assay using BHK-21 cells.

**Table 1:** Primer sequences used for RT-qPCR.

	Forward primer sequence (5′- > 3′)	Reverse primer sequence (5′- > 3′)
POWV Probe	56-FAM/TGGCATCCG/Zen/AGAAAGTGATCCTGC/3IABkFQ	
POWV	GGCTGCAAATGAGACCAATTC	CAGCGACACATCTCCATAGTC

### 2.4 Histopathology

At day 3 post inoculation, we harvested the tissues after cardiac perfusion with 1X PBS and fixed them in 4% paraformaldehyde (PFA). Optimal cutting temperature (OCT) medium was used for embedding tissues (Tissue-Plus™, Cat# 23–730-571). OCT-embedded tissues were sectioned and stained with hematoxylin and eosin (H&E) for histopathological evaluation (Abcam, Cat# ab245880) (Stone et al., 2025). Tissue sections were stained with DsRNA (J2) mouse antibody (Absolute Antibody, Cat# Ab01299-2.0) overnight at 4 °C, followed by incubation with Alexa Fluor 488 Anti-mouse (Invitrogen, Cat# A10684) antibody for 30 min at room temperature. Additionally, brain sections were incubated with GFAP-Alexa Fluor 594 Anti-mouse (Cell Signaling, Cat# 8152). Spleen, liver and kidney tissue sections were stained with CD68-Alexa Fluor^®^ 488 (Cell Signaling Technology, Cat# 51644) overnight at 4 °C. We mounted the stained sections with Prolong™ Glass Antifade Mountant with NucBlue™ StainDAPI (Thermo Fisher Scientific, Cat# P36981). Images were acquired using the Invitrogen™-EVOS™ M5000 Cell Imaging System and analyzed using ImageJ software (Elsharkawy et al., 2025a,b; Ge et al., 2025).

### 2.5 Luminex assay

Tissues harvested from POWV-infected and mock-infected mice were homogenized in 1X PBS with protease inhibitor in the bullet blender (Next Advanced). Homogenates and serum were tested using the MILLIPLEX MAP Mouse Cytokine/Chemokine Magnetic Bead Panel - Premixed 25-Plex - Immunology Multiplex Assay (Cat# MCYTMAG-70 K-PX25) as per manufacturer instructions. We calculated the sample concentrations using the Belysa^®^ Immunoassay Curve Fitting Software (Millipore Sigma) (Basu et al., 2024; Browne et al., 2025; Oh et al., 2024).

### 2.6 Flow cytometry

For the cytometric analysis of the spleens, we anesthetized mice using isoflurane, followed by 1X PBS cardiac perfusion. Spleen single-cell suspensions were generated using the gentle MACS tissue dissociator (Miltenyi Biotec, Cat#130-093-235). We incubated the spleen single-cell suspensions with Fc Block antibody (BD Biosciences) in BD FACS™ Pre-Sort Buffer (BD Biosciences) for 10 min at room temperature before staining, followed by fixable Viability Stain 575 V (BD Biosciences). Next, we incubated the samples with antibodies against the following markers: FITC Rat Anti-Mouse CD45 (BD, Cat# 553080), PE Rat Anti-Mouse CD4 (BD, Cat# 553730), PerCP-Cy5.5 Rat Anti-Mouse CD8β (BD, Cat# 567597), and APC-Cy7 Rat Anti-Mouse CD19 (BD, Cat# BDB561737) or FITC Rat Anti-Mouse CD45 (BD, Cat# 553080), APC Rat Anti-Mouse CD11b (BD, Cat# 553312) and PE Rat Anti-Mouse Ly6C (BD, Cat# 568954). Cells were stained for 30 min on ice, then washed and fixed in fixation buffer (eBioscience). We acquired flow cytometry data on a BD LSRFortessa™ Cell Analyzer and used the FlowJo software for further analysis (Elsharkawy et al., 2024; Kumar et al., 2015).

### 2.7 Statistical analysis

We performed the statistical analyses using the GraphPad Prism software, version 10 and considered results statistically significant at *p*-values of *p* < 0.05. We used unpaired Student’s *t*-test to compare the two groups. We used one-way analysis of variance (ANOVA) followed by Tukey’s multiple comparisons test, non-parametric Kruskal–Wallis test followed by Šídák’s multiple comparisons test or mixed effects analysis followed by Fisher’s LSD to compare multiple groups.

## 3 Results

### 3.1 Enhanced mortality in *Ifnar^−/−^* mice following POWV infection

**Figure 1:**
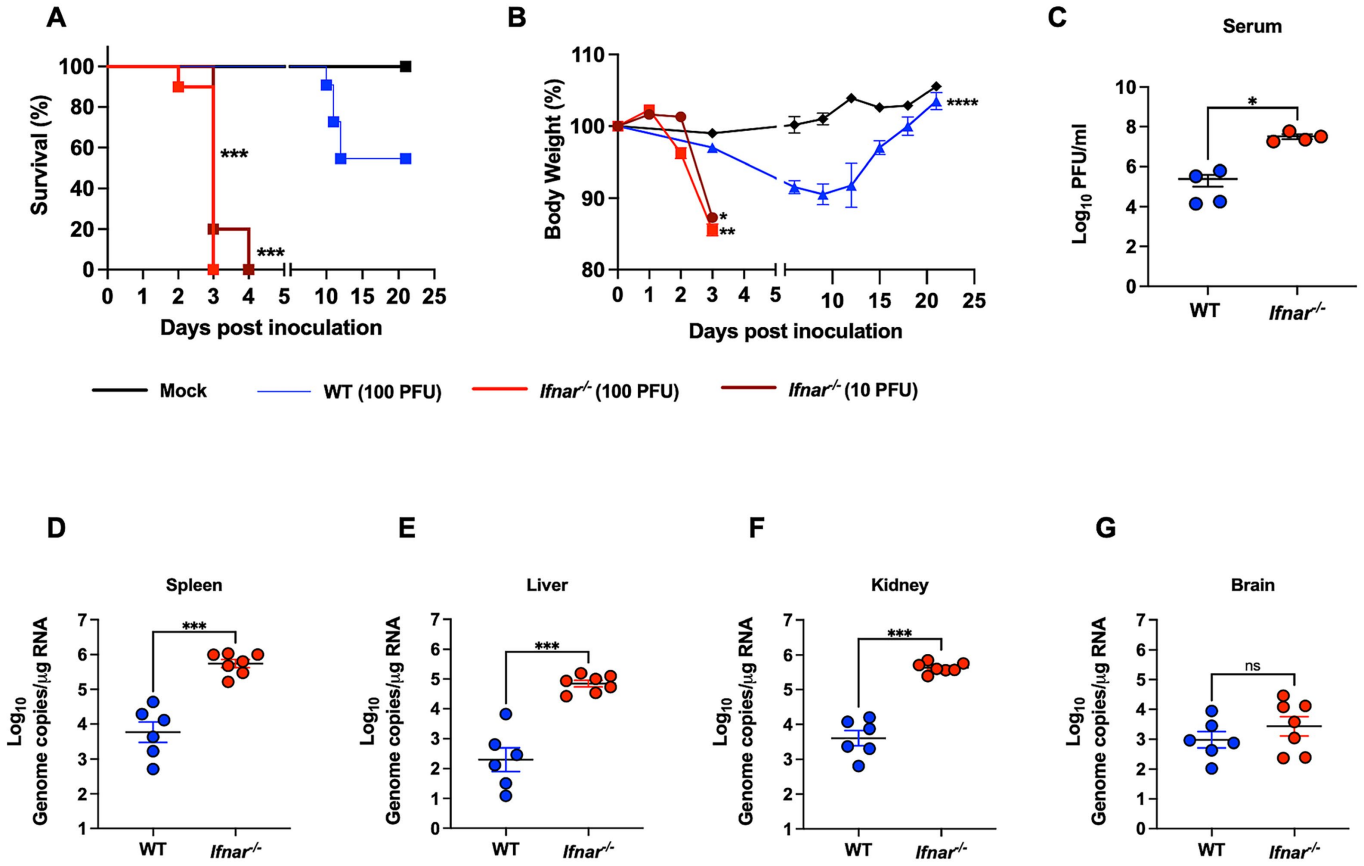
Morbidity, mortality, and viral load in WT and in *Ifnar^−/−^* mice following POWV infection. **(A)** Kaplan–Meier survival curve was generated for mock-infected mice (*n* = 3), POWV-infected WT mice (*n* = 6), and POWV-infected *Ifnar^−/−^* (*n* = 10 for each infectious dose). The Log-rank (Mantel-Cox) test showed statistical significance differences in survival between WT-infected and *Ifnar^−/−^*-infected at both infectious doses (*p*-value < 0.0001). **(B)** Weight loss for mock-infected mice, WT-infected mice (100 PFU), *Ifnar^−/−^*-infected (10 PFU and 100 PFU). Mice were weighed daily for 21 days post infection. Weights are expressed as percentage of initial body weight prior to infection (day 0). Weight change curves for *Ifnar^−/−^*-infected (10 PFU, 100 PFU, *n* = 10), WT-infected mice (100 PFU, *n* = 6), and mock-infected mice groups (*n* = 3). Mixed effects analysis (*p < 0.05, **p < 0.01, ***p < 0.001, ****p < 0.0001). **(C)** Infectious virus titers in the serum are plotted for each mouse at 3 dpi; middle horizontal bar indicates the mean and error bars are SEM. *p*-values were calculated by Mann–Whitney test (*p*-value = 0.0143; *n* = 3 for mock-infected; *n* = 4 for POWV-infected). **(D–G)** Viral RNA load in mock- and POWV-infected tissues. Mice were inoculated with PBS (mock) or 100 PFU of POWV. Brain, spleen, liver, and kidney were harvested at 3 dpi and viral load was determined via RT-qPCR. Viral load data is expressed as genome copies per microgram of RNA after normalization to a standard curve. Data is plotted for each mouse tissue; Horizontal bars indicate mean values for the group and error bars are SEM. Data are presented on a Log10 scale. Statistical significance was determined by ordinary one-way ANOVA followed by Šídák’s multiple comparisons test (*p < 0.05, **p < 0.01, ***p < 0.001, ****p < 0.0001; *n* = 6–7).

In this study we used mice deficient in the IFNα/β receptor (*Ifnar^−/−^*) to determine its role in POWV pathogenesis. Six-week-old *Ifnar^−/−^* and C57BL/6J (WT) mice were infected subcutaneously by the footpad with a low dose of POWV LB strain. We infected *Ifnar^−/−^* mice with 100 PFU or 10 PFU and WT mice with 100 PFU. Mice were monitored daily for signs of disease including rounded posture, piloerector fur, and gait abnormalities. As early as day 2 post infection (dpi), *Ifnar^−/−^* mice infected with 100 PFU started losing significant body weight. At 3 dpi, *Ifnar^−/−^* mice showed severe signs of illness regardless of dose given and all mice succumbed to infection by 4 dpi. In contrast, WT mice did not show signs of disease or weight loss until 7–8 dpi. Approximately 54% of infected WT mice succumbed to infection between 10 and 12 dpi (Figures 1A,B).

We next investigated the virus replication kinetics and tissue tropism in WT and *Ifnar^−/−^* mice. Mice were infected with 100 PFU of POWV and euthanized at 3 dpi. We quantified viremia in the serum by plaque assay. At 3 dpi, *Ifnar^−/−^* mice had significantly higher levels of infectious virus titers (mean = 3.2 × 10^7^) in the serum compared to WT mice (mean = 2.4 × 10^5^) (Figure 1C). We also analyzed viral load in peripheral organs by RT-qPCR. Compared to the viral load in WT mice (mean = 5 × 10^3^), *Ifnar^−/−^* mice had significantly higher levels of viral load (mean = 5 × 10^5^) in the spleen (Figure 1D). Next, we determined the viral load in infected livers. *Ifnar^−/−^* mice had significantly higher levels of viral load (mean = 6.3 × 10^4^) in the liver compared to WT mice (mean = 1.9 × 10^2^) (Figure 1E). Similarly, we detected significantly increased viral load in the kidney of *Ifnar^−/−^* mice (mean = 3.9 × 10^5^) compared to WT mice (mean = 3.9 × 10^3^) (Figure 1F). Notably, *Ifnar^−/−^* mice also had slightly higher viral burden in the brain compared to WT mice; however, the difference was not statistically significant (mean = 2.5 × 10^3^ and 7.9 × 10^2^, respectively) (Figure 1G).

### 3.2 Detection of dsRNA in tissues following POWV infection

**Figure 2:**
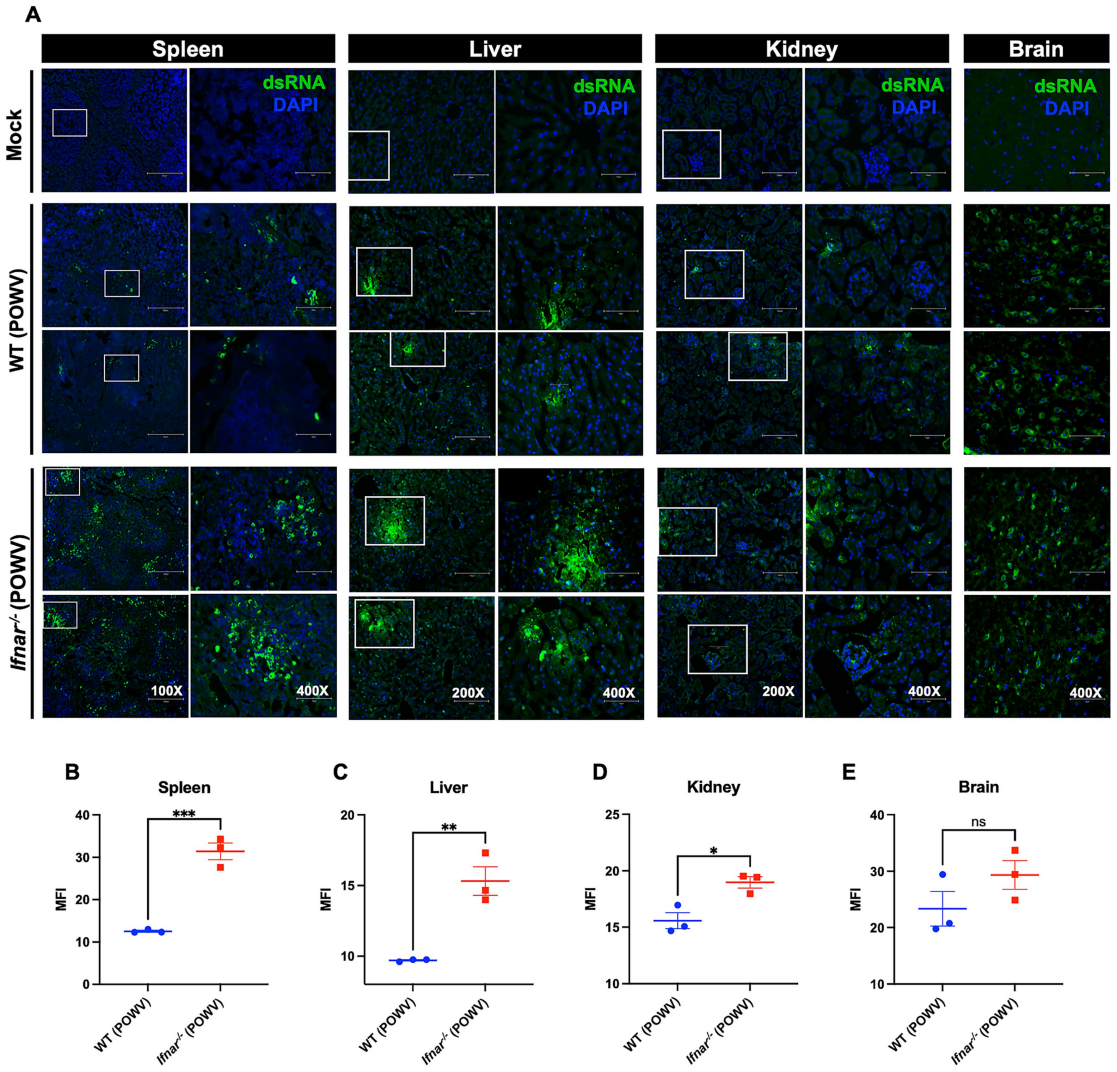
Detection of dsRNA in tissues following POWV infection. **(A)** Tissues were collected from mock- and POWV-infected mice at 3 dpi. Collected tissues stained with dsRNA (green) and DAPI (blue). Representative images of spleen (scale bars are 250 μm on original images and 75 μm on enlarged images), liver (scale bars are 150 μm on original images and 75 μm on enlarged images), kidney (scale bars are 150 μm on original images and 75 μm on enlarged images), and brain (scale bars are 75 μm). **(B–E)** Mean fluorescence intensity (MFI) was determined for dsRNA-stained sections by ImageJ. Middle horizontal bar indicates the mean and error bars are SEM. Statistical significance was determined by unpaired *t*-test (*p < 0.05, **p < 0.01, ***p < 0.001; *n* = 3).

Detection of double-stranded RNA (dsRNA) by immunofluorescence is a marker of active flavivirus replication within infected tissues (Mazeaud et al., 2018; O’Brien et al., 2015; Son et al., 2015). We next assessed dsRNA presence by immunofluorescence staining. Consistent with viral load measurements, *Ifnar^−/−^* mice displayed markedly stronger dsRNA signal in the spleen compared to WT controls (Figures 2A,B). In the liver, dsRNA was detected in both strains but was more pronounced in *Ifnar^−/−^* mice (Figures 2A,C). Similarly, kidney tissue from *Ifnar^−/−^* mice exhibited higher dsRNA levels relative to WT mice (Figures 2A,D). In contrast, brain tissue from both *Ifnar^−/−^* and WT mice showed robust dsRNA staining at comparable levels, in line with the viral load data (Figures 2A,E). Quantitative analysis showed significant differences in mean fluorescence intensity (MFI) of dsRNA signal between WT and Ifnar^−/−^ spleens, liver and kidney.

### 3.3 Systemic inflammation in *Ifnar^−/−^* mice following POWV infection

**Figure 3:**
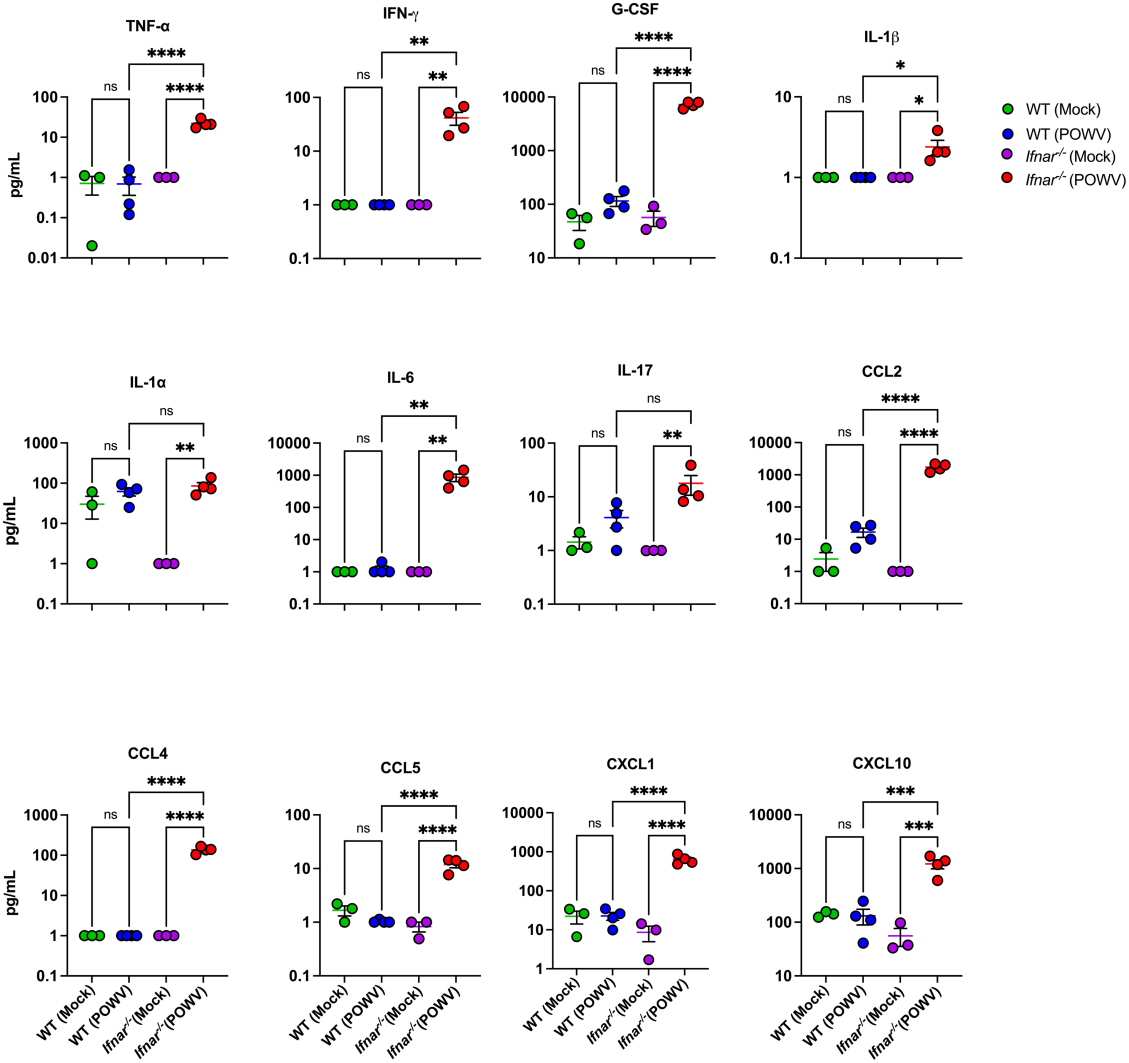
Cytokine and chemokine protein levels in the serum following POWV infection. Mice were inoculated with PBS (mock) or 100 PFU of POWV. Serum was collected at 3 dpi. Cytokine and chemokine protein levels were measured by a multiplex immunoassay. Each data point represents an individual mouse. Middle horizontal bar indicates the mean and error bars are SEM. Data are presented on a Log10 scale and each analyte is plotted on an independent scale. *p*-values were calculated by One-way ANOVA followed by Šídák’s multiple comparisons test (*p < 0.05, **p < 0.01, ***p < 0.001, ****p < 0.0001; *n* = 3 for mock-infected mice; *n* = 4 for POWV-infected mice).

Next, we evaluated systemic inflammation by measuring proteins levels of key cytokines and chemokines in the serum using a multiplex immunoassay. We did not observe significant increase in inflammatory mediators in the WT mice at 3 dpi when compared to mock-infected controls. In contrast, POWV-infected *Ifnar^−/−^* mice exhibited significant increase the levels of several proinflammatory mediators. We detected significantly high levels of TNF-α (mean = 22.1 pg./mL) and IL-6 (mean = 861.3 pg./mL). Granulocyte colony-stimulating factor (G-CSF) levels were also significantly increased in *Ifnar^−/−^* mice (mean = 7227.7 pg./mL). Additionally, we detected a significant increase in the levels of several chemokines including C–C motif (CCL2 and CCL5, mean = 1731.1 and 11.89 pg./mL, respectively) and C–X–C motif (CXCL1 and CXCL10, mean = 639.3 and 1218.9 pg./mL, respectively) in the serum collected from POWV-infected *Ifnar^−/−^* mice (Figure 3). Overall, these findings show that the absence of IFNα/β receptor signaling accelerates mortality and induces an exacerbated systemic inflammatory response following POWV infection in mice.

### 3.4 Pathology in the peripheral organs of WT and *Ifnar^−/−^* mice following POWV infection

**Figure 4:**
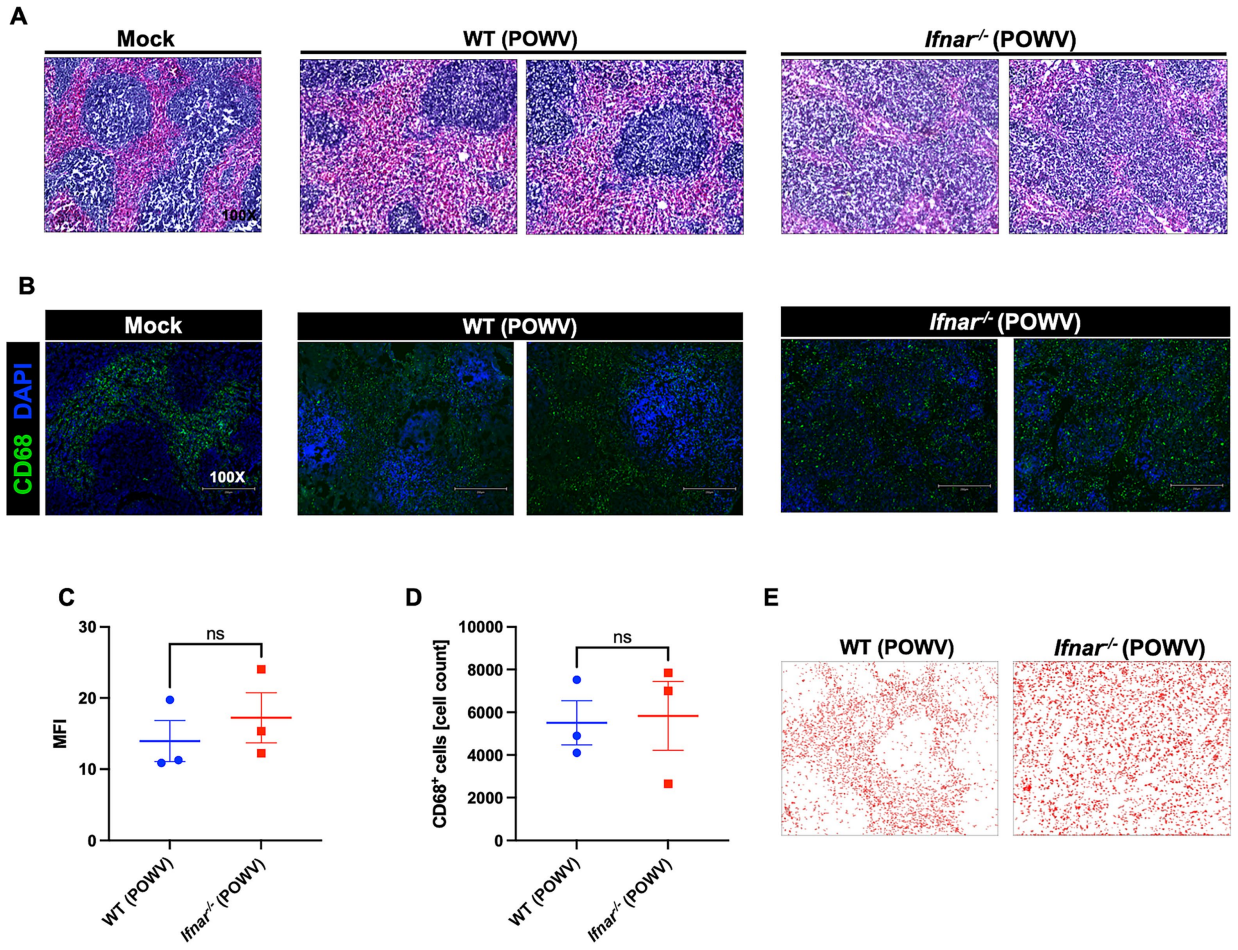
Spleen pathology following POWV infection. **(A)** H&E staining of spleen tissue harvested at 3 dpi. **(B)** Microscopic imaging of mouse spleens stained with CD68-Alexa Fluor^®^ 488 (green) and DAPI (blue). Scale bars are 250 μm. **(C)** MFI was determined for CD68-stained sections by ImageJ. **(D)** Number CD68-positive cells in the spleen using ImageJ. Middle horizontal bar indicates the mean and error bars are SEM (*n* = 3/group). Statistical significance was determined by unpaired *t*-test. **(E)** ImageJ-generated visualization of CD68-positive cell distribution in the spleen.

The spleen is a primary organ in the lymphatic system and plays a crucial role during viral infection. Therefore, we assessed POWV-induced pathology using H&E staining of spleen tissues collected at 3 dpi. Compared to WT, *Ifnar^−/−^* spleen had significantly altered morphology with loosely distributed white and red pulps (Figure 4A). To evaluate the localization of splenic macrophages, we deployed CD68 labeling of infected spleens collected at 3 dpi (Figure 4B). Quantitative analysis showed no significant differences in MFI or total CD68-positive cell counts between WT and Ifnar−/− spleens (Figures 4C,D). Traditionally, CD68 is observed in the red pulp, the distribution of CD68^+^ splenic macrophages in infected *Ifnar^−/−^* mice was markedly altered compared to mock-infected and WT POWV-infected spleen (Figure 4E).

**Figure 5:**
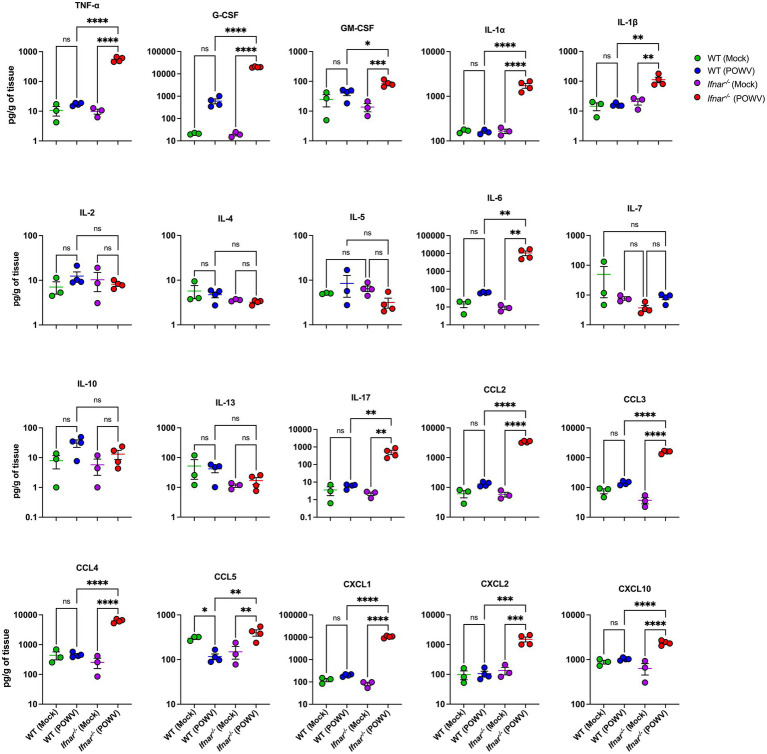
Splenic inflammatory response following POWV infection. Cytokine and chemokine protein levels were measured by a multiplex immunoassay. Each data point represents an individual mouse. Middle horizontal bar indicates the mean and error bars are SEM. Data are presented on a Log10 scale and each analyte is plotted on an independent scale. *p*-values were calculated by mixed effects analysis followed by Fisher’s LSD (*p < 0.05, **p < 0.01, ***p < 0.001, ****p < 0.0001; *n* = 3 for mock-infected mice; *n* = 4 for POWV-infected mice).

We also investigated the inflammatory response in the spleen using a multiplex immunoassay. Compared to mock-infected controls, spleens from POWV-infected WT mice showed no significant differences. On the other hand, spleens from POWV-infected *Ifnar^−/−^* mice showed significant upregulation in multiple pro-inflammatory cytokines and chemokines. Compared to WT, *Ifnar^−/−^* had significantly higher levels of TNF-α, IFN-γ, G-CSF, GM-CSF, IL-1α, IL-1β, IL-6, and IL-17. No differences were detected in the levels of IL-2, IL-10, and IL-13 between mock- and POWV-infected tissue in both WT and *Ifnar^−/−^* mice. Furthermore, levels of CCL2, CCL3, CCL4, CXCL1, CXCL2 and CXCL10 were significantly higher in infected *Ifnar^−/−^* spleens compared to WT spleens (Figure 5).

**Figure 6:**
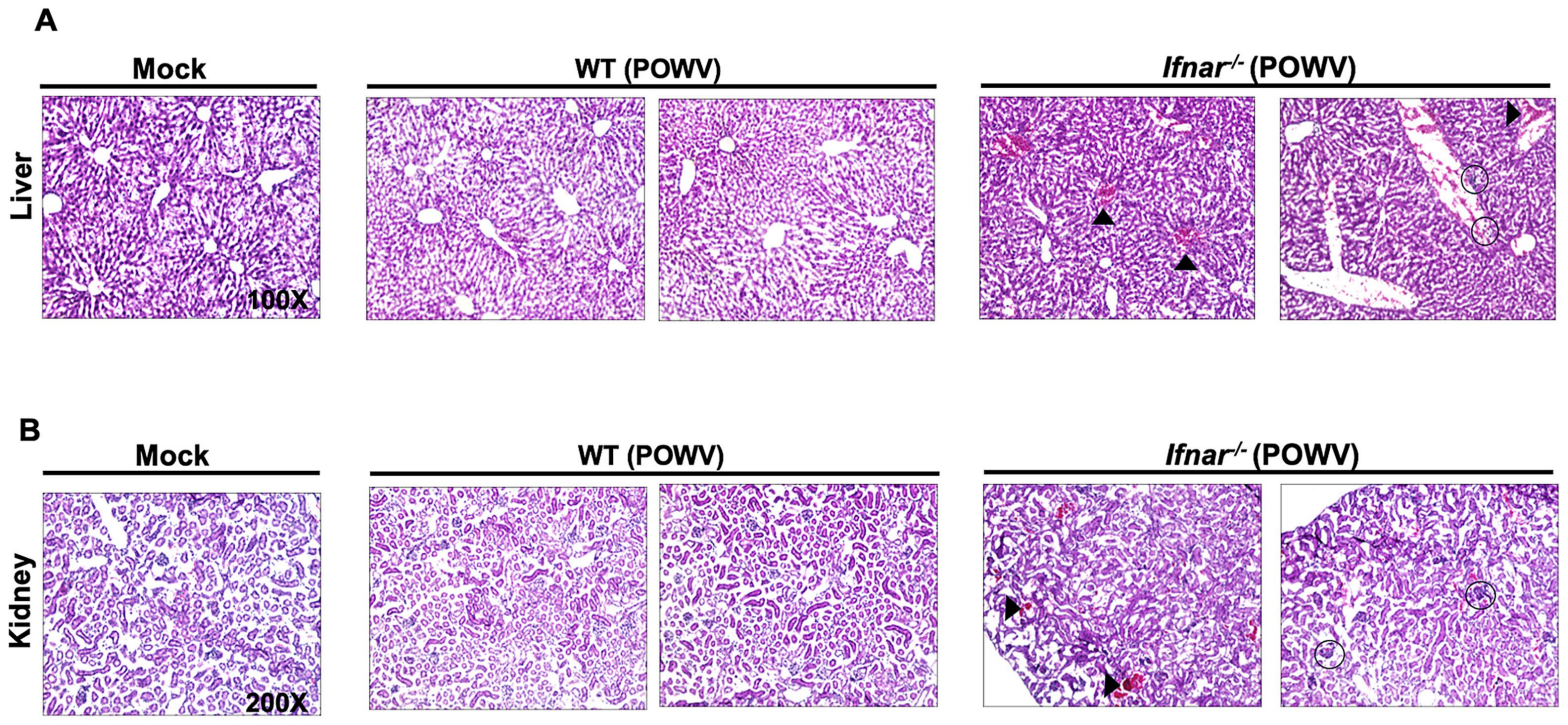
Pathology in the liver and in the kidney following POWV infection. Liver and kidney tissues were harvested from mock- or POWV-infected mice. **(A)** H&E staining of liver tissue harvested at 3 dpi. Arrow heads indicate congestion of central veins; circles indicate infiltration of inflammatory cells. **(B)** H&E staining of kidney tissue harvested at 3 dpi. Arrow heads indicate congestion of hemorrhagic sites; circles indicate congestion of congestion of glomerular tuft. *n* = 3 for mock-infected mice; *n* = 4 for POWV-infected mice.

Next, we examined POWV-induced pathology in the liver and in the kidney. Histological analysis showed hepatocyte swelling, infiltration of inflammatory cells, and congestion of central veins in infected *Ifnar^−/−^* mice. In contrast, no significant pathology was detected in the livers of POWV-infected WT mice (Figure 6A). We observed several hemorrhagic sites and congestion of glomerular tuft in infected *Ifnar^−/−^* kidney (Figure 6B).

**Figure 7:**
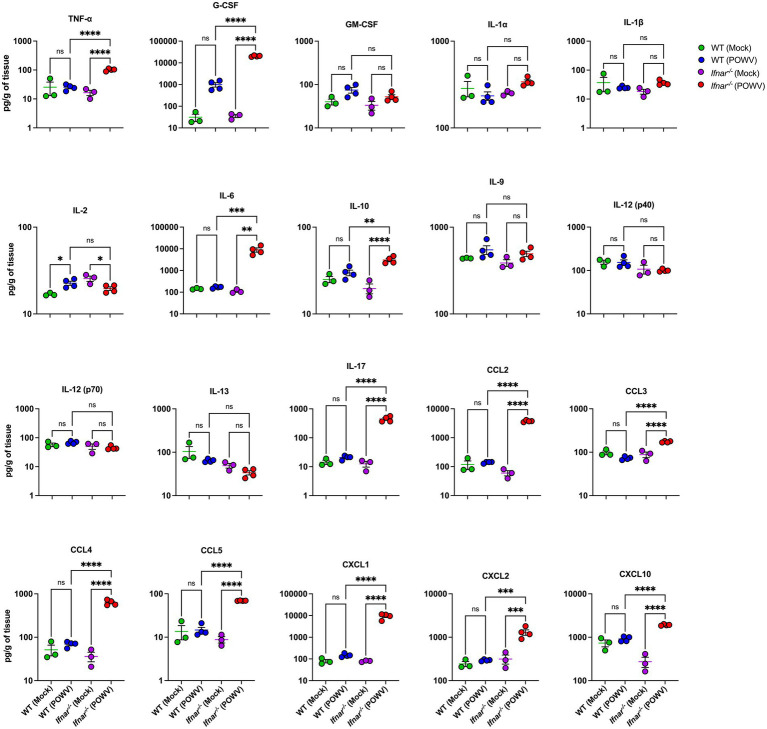
Inflammation in the liver following POWV infection. Cytokine and chemokine protein levels were measured in the liver at 3 dpi. Each data point represents an individual mouse. Middle horizontal bar indicates the mean and error bars are SEM. Data are presented on a Log10 scale and each analyte is plotted on an independent scale. *p*-values were calculated by mixed effects analysis followed by Fisher’s LSD (*p < 0.05, **p < 0.01, ***p < 0.001, ****p < 0.0001; *n* = 3 for mock-infected mice; *n* = 4 for POWV-infected mice).

Further, we assessed inflammatory response in the liver and in the kidney following POWV infection. Compared to infected WT liver, infected *Ifnar^−/−^* liver had significantly higher protein levels of TNF-α, G-CSF, IL-6, IL-10, IL-17, CCL2, CCL3, CCL4, CCL5, CXCL1, CXCL2, and CXCL10 (Figure 7). For example, we detected significantly higher levels of TNF-α in *Ifnar^−/−^* mice following POWV infection (mean = 102, 16.4 pg./g in POWV- and mock-infected *Ifnar^−/−^* liver, respectively). Similarly, we detected significantly increased levels of IL-6 in *Ifnar^−/−^* mice (mean = 8,979, 111 pg./g in POWV- and mock-infected Ifnar^−/−^ liver, respectively). On the other hand, we did not detect any significant differences in IL-1α, IL-1β, IL-9, IL-12 and IL-13 levels in POWV-infected WT and *Ifnar^−/−^* mice compared to mock-infected controls. Additionally, we detected a significant increase in the levels of several chemokines including CCL2 and CCL4 (mean = 3,738, 631 pg./g, respectively) and CXCL1 and CXCL10 (mean = 9392.7 and 1917.1 pg./g, respectively) in POWV-infected *Ifnar^−/−^* mice.

**Figure 8:**
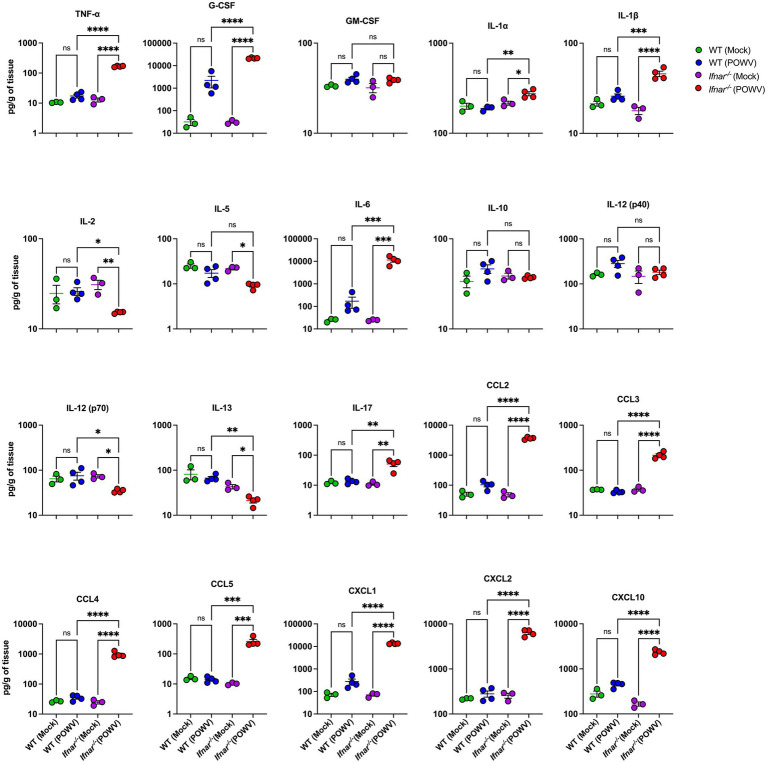
Cytokine and chemokine levels in the kidney following POWV infection. Cytokine and chemokine protein levels were measured in the kidney at 3 dpi. Each data point represents an individual mouse. Middle horizontal bar indicates the mean and error bars are SEM. Data are presented on a Log10 scale and each analyte is plotted on an independent scale. *p*-values were calculated by mixed effects analysis followed by Fisher’s LSD (*p < 0.05, **p < 0.01, ***p < 0.001, ****p < 0.0001; *n* = 3 for mock-infected mice; *n* = 4 for POWV-infected mice).

Compared to WT mice, *Ifnar^−/−^* mice had significantly higher levels of TNF-α, G-CSF, IL-1α, IL-1β, IL-6, IL-17, CCL2, CCL3, CCL4, CCL5, CXCL1, CXCL2, and CXCL10 in the kidney. For example, we detected higher levels of IL-1β in *Ifnar^−/−^* mice following POWV infection (mean = 46, 18 pg./g in POWV- and mock-infected *Ifnar^−/−^* kidneys, respectively). On the other hand, we did not detect significant increase in IL-1β levels in WT mice following POWV infection (mean = 25.8, 21.3 pg./g in POWV- and mock-infected WT kidneys, respectively). Similarly, we detected significantly increased levels of TNF-α in *Ifnar^−/−^* mice (mean = 167, 13 pg./g in POWV- and mock-infected Ifnar^−/−^ kidneys, respectively). We detected a slight increase in TNF-α levels in POWV-infected WT mice with a mean of 17.2 pg./g compared to mock-infected controls with a mean of 10.4 pg./g. However, the difference was not statistically significant. In contrast, the levels of anti-inflammatory cytokine IL-2 were significantly reduced in *Ifnar^−/−^* kidney in comparison with WT kidney (mean = 15.2, 26 pg./g, respectively). Similarly, we detected a significant decrease in the levels of IL-13 in *Ifnar^−/−^* compared to WT kidney (mean = 21.3, 66.6 pg./g, respectively) (Figure 8). Similar to the spleen and liver, levels of various chemokines such as CCL2, CCL3, CCL4, CCL5, CXCL1, CXCL2 and CXCL10 were significantly higher in infected *Ifnar^−/−^* kidneys compared to WT kidneys.

### 3.5 Flow cytometric analysis of splenic immune cells following POWV infection

**Figure 9:**
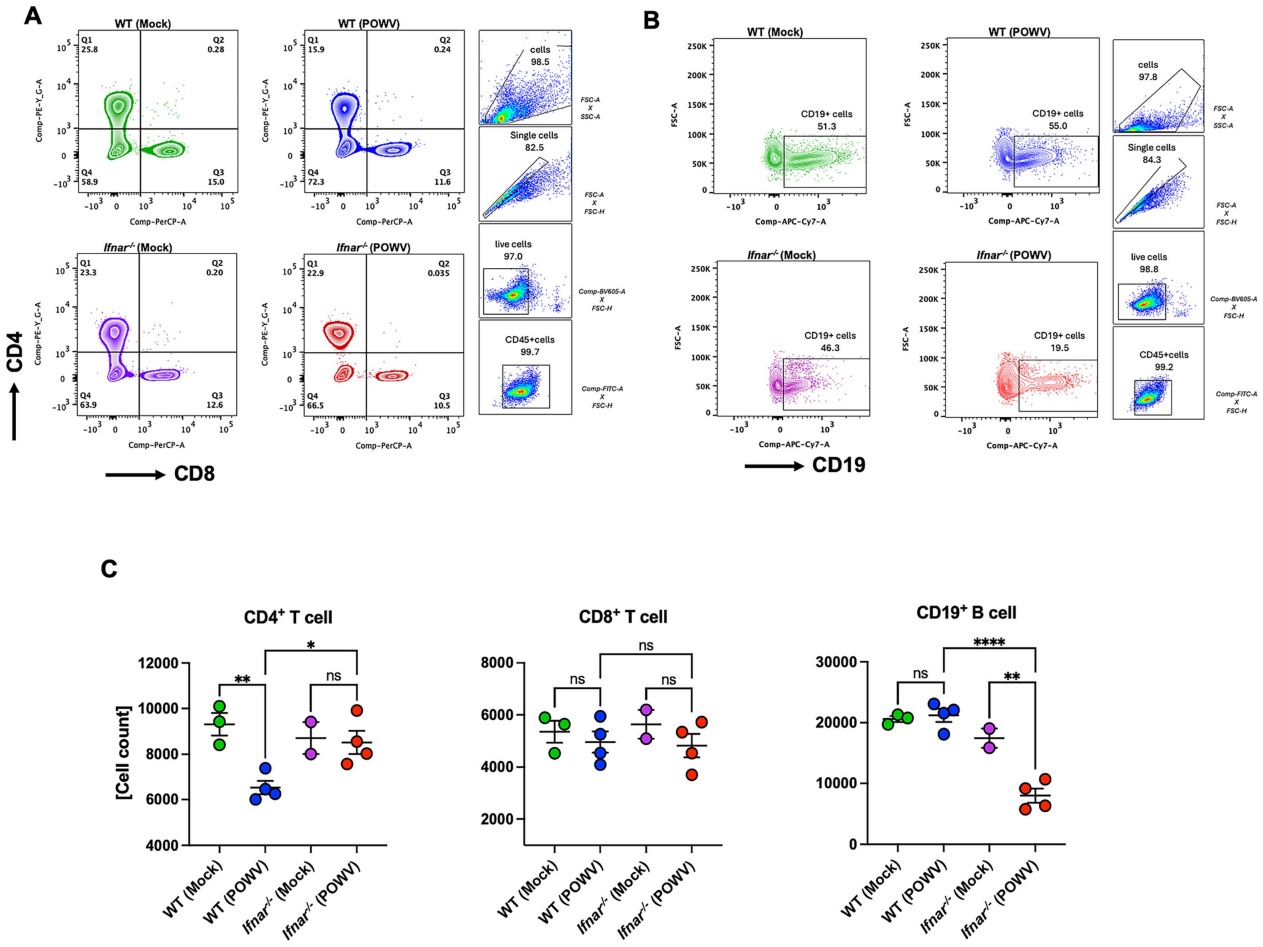
Flow cytometric analysis of T and B cell responses in the spleen following POWV infection. **(A)** Gating strategy and FACS plots of CD4^+^ and CD8^+^ T cells in the spleen. **(B)** Gating strategy and FACS plots of CD19^+^ cells. **(C)** CD4^+^ T, CD8^+^ T, and CD19^+^ cell numbers in the spleen. Each point represents an individual mouse. The bars indicate the mean and error bars are SEM. Ordinary one-way ANOVA, followed by Tukey’s multiple comparisons test (*p < 0.05, **p < 0.01, ***p < 0.001, ****p < 0.0001; *n* = 2–3 for mock-infected mice; *n* = 4 for POWV-infected mice).

**Figure 10:**
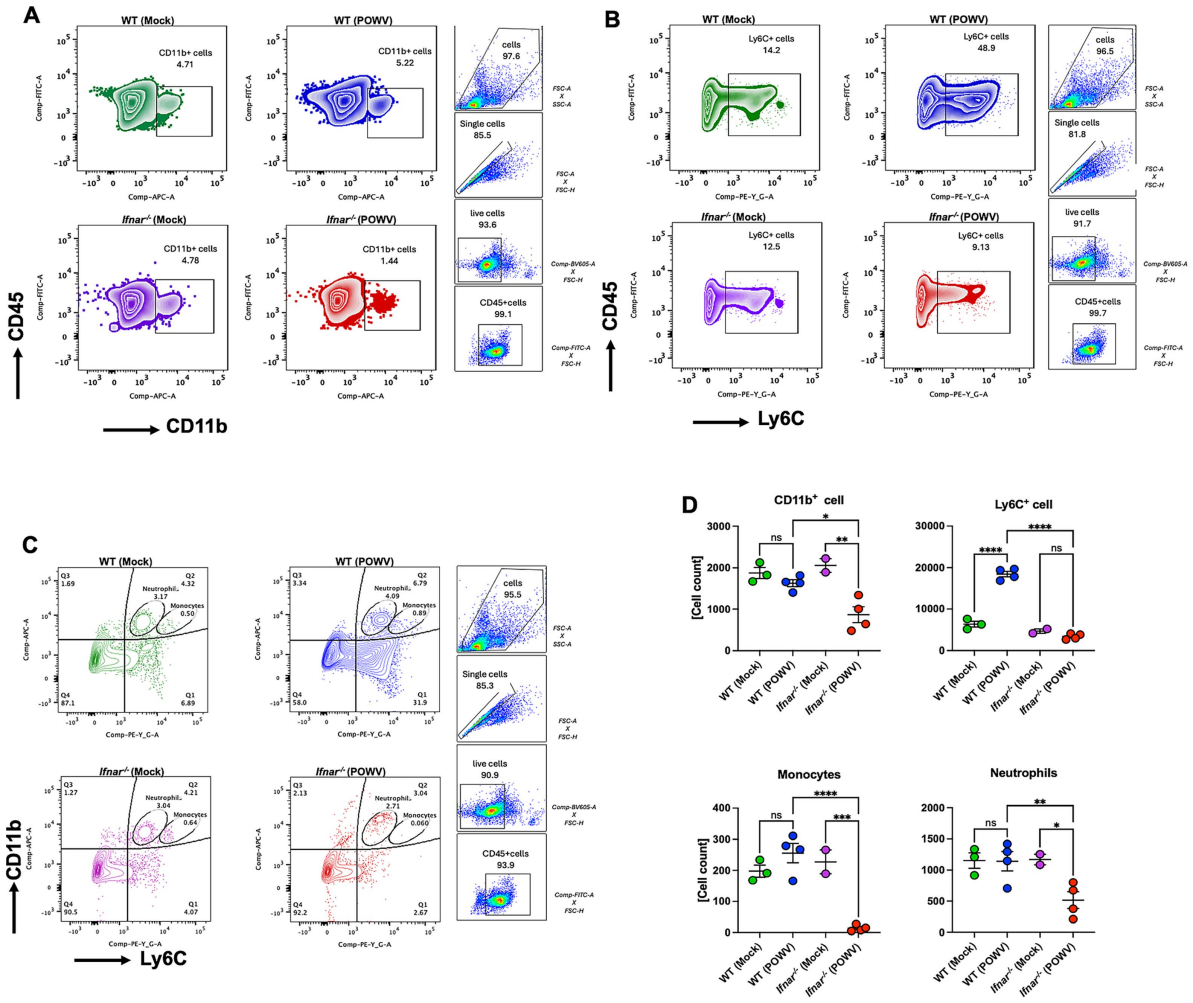
Cellular immune response in the spleen following POWV infection. Flow cytometric analysis of spleen following inoculation with POWV or mock infection. **(A)** Gating strategy and FACS plots of CD11b^+^ cells. **(B)** Gating strategy and FACS plots of Ly6C^+^ cells in the spleen. **(C)** Gating strategy and FACS plots of CD11b^+^ Ly6C^+^ cells in the spleen. **(D)** CD11b^+^, Ly6C^+^, neutrophils and monocytes numbers in the spleen. Each point represents an individual mouse. The bars indicate the mean and error bars are SEM. Ordinary one-way ANOVA, followed by Tukey’s multiple comparisons test (*p < 0.05, **p < 0.01, ***p < 0.001, ****p < 0.0001; *n* = 2–3 for mock-infected mice; *n* = 4 for POWV-infected mice).

To explore differences in splenic cellular immune response, we performed flow cytometric analysis of spleens collected at 3 dpi. In POWV-infected WT mice, we observed a significant reduction in CD4^+^ T cells. Interestingly, no reduction in CD4^+^ T cells was observed in *Ifnar^−/−^* mice. On the other hand, CD8^+^ T cells did not show any significant differences between POWV- and mock-infected in either mouse strains (Figures 9A,C). We also investigated B cell response in the spleen following POWV infection. While no significant changes were detected in B cell response in POWV-infected WT mice, a significant reduction in B cell number was detected in the spleen of *Ifnar^−/−^* mice following POWV infection (Figures 9B,C). Interestingly, POWV infection resulted in significant decrease in the number of splenic CD11b^+^ cells in *Ifnar^−/−^* mice. No significant difference in CD11b^+^ cell count was observed between POWV- and mock-infected WT mice (Figures 10A,D). POWV infection also caused a marked increase in the number of Ly6C^+^ cells in POWV-infected WT spleens. In contrast, we did not detect any significant increase in the number of Ly6C^+^ cells in POWV-infected *Ifnar^−/−^* spleens (Figures 10B,D). The number of monocytes increased slightly in WT-infected spleens, but the difference was not statistically significant. In contrast, infected *Ifnar^−/−^* spleens had significantly reduced numbers of monocytes following POWV infection (Figures 10C,D). Similarly, the number of neutrophils decreased significantly in *Ifnar^−/−^* spleens following POWV infection while no difference was detected between POWV- and mock-infected WT spleens (Figures 10C,D).

### 3.6 Neuroinflammation in *Ifnar^−/−^* mice following POWV infection

**Figure 11:**
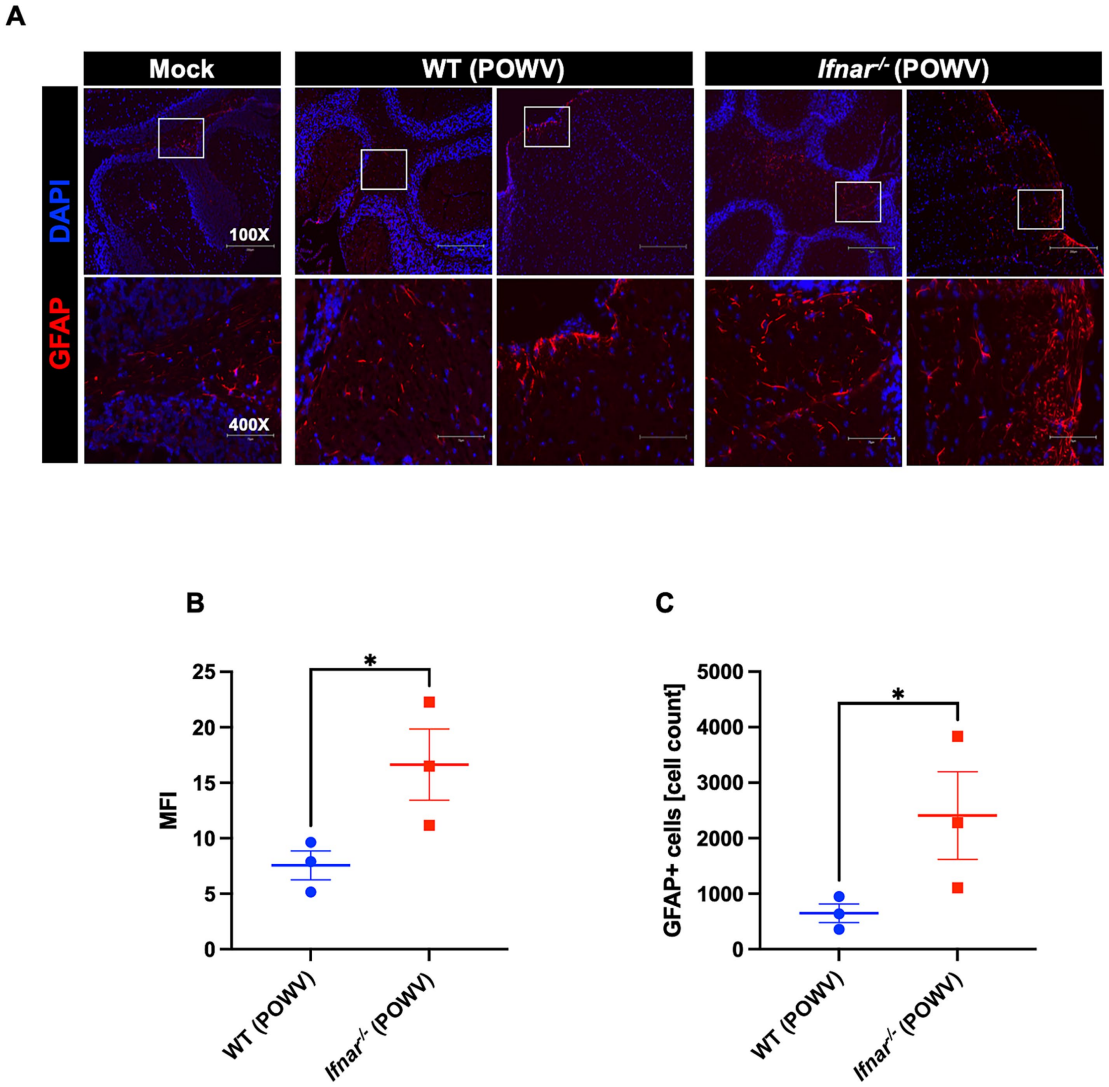
GFAP expression in the brain following POWV infection. **(A)** Microscopic imaging of mouse brains stained with GFAP-Alexa Fluor 594 Anti-mouse (red) and DAPI (blue). Scale bars are 250 μm on original images and 75 μm on enlarged images. **(B)** MFI was determined for GFAP-stained sections by ImageJ. **(C)** Number GFAP-positive cells in the brain using ImageJ. Middle horizontal bar indicates the mean and error bars are SEM (*n* = 3/group). Statistical significance was determined by unpaired t test (*p < 0.05).

POWV has classically been known as a neurotropic virus and death in wild-type mice typically follows neuroinvasion. Flaviviruses have been shown to trigger astrocyte activation which is characterized by morphological changes and altered expression of several genes such as the glial fibrillary acidic protein (GFAP) (Pekny and Pekna, 2016; Potokar et al., 2019). Therefore, we assessed the expression of GFAP, a marker for astrocyte activation, in the brain of POWV-infected WT and *Ifnar^−/−^* mice. Immunofluorescence analyses revealed higher GFAP expression in *Ifnar^−/−^* infected brains, mainly in the cerebellum and in the meninges (Figure 11A). Similarly, quantitative analysis showed significant differences in MFI and total GFAP-positive cell counts between WT and *Ifnar^−/−^* spleens (Figures 11B,C).

**Figure 12:**
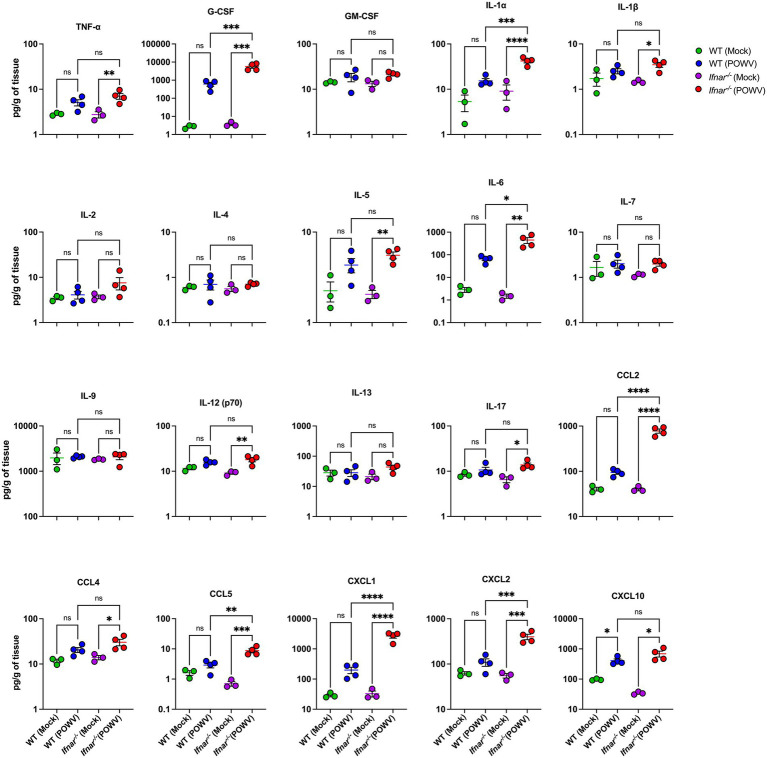
Neuroinflammation following POWV infection. Cytokine and chemokine protein levels were measured in the brain at 3 dpi. Each data point represents an individual mouse. Middle horizontal bar indicates the mean and error bars are SEM. Data are presented on a Log10 scale and each analyte is plotted on an independent scale. *p*-values were calculated by One-way ANOVA followed by Šídák’s multiple comparisons test (*p < 0.05, **p < 0.01, ***p < 0.001, ****p < 0.0001; *n* = 3 for mock-infected mice; *n* = 4 for POWV-infected mice).

We also assessed local CNS inflammation through cytokine/chemokine protein levels measurement. Despite the insignificant difference in brain viral load between WT and *Ifnar^−/−^* mice, we detected considerably higher levels of proinflammatory cytokines and chemokines in POWV-infected *Ifnar^−/−^* brains. Compared to POWV-infected WT, *Ifnar^−/−^* mice had significantly higher levels of G-CSF, IL-1*α*, IL-6, CCL2, CCL5, CXCL1, and CXCL2. Compared to mock-infected controls, both POWV-infected WT and *Ifnar^−/−^* brains had significantly increased levels of CXCL10 (mean = 408, 691 pg./g for POWV-infected WT and *Ifnar^−/−^* brains, respectively) (Figure 12).

## 4 Discussion

Type-I interferon response is one of the most potent antiviral mechanisms against flaviviruses (Daffis et al., 2009; O’Neill and Bowie, 2010; Weber et al., 2014). While it has been shown that type-I IFN signaling plays a critical role in mosquito-borne flavivirus infection (Daffis et al., 2009; Keller et al., 2006), its role during a tick-borne POWV infection remains to be fully elucidated. In this study, we demonstrated that POWV infection in mice lacking a functional IFN-α/β receptor (*Ifnar^−/−^*) mice resulted in a rapidly fatal infection. Our findings showed that type-I interferon signaling is essential for the early control of POWV replication. In the absence of type-I IFN signaling, mice exhibited elevated viremia and increased viral loads in the peripheral tissues. This deficiency also impaired the immune response in the spleen and was associated with heightened systemic inflammation and severe pathology in peripheral organs. Notably, while IFN-α/β receptor deficiency did not significantly affect viral burden in the brain, it led to an exaggerated inflammatory response in the brain.

Type-I interferon receptors knockout mice (*Ifnar^−/−^*) are more susceptible to viral infections due to the impaired immune response. Therefore, *Ifnar^−/−^* mice have been commonly used to model disease caused by highly pathogenic viruses. Type-I IFN-mediated response is necessary to resist Zika virus (ZIKV) replication (Gobillot et al., 2020). Mice lacking type-I IFN signaling are highly susceptible to ZIKV and succumb to infection by day 10 after infection (Lazear et al., 2016). Mice infected with POWV spooner strain, a less lethal strain than the LB strain showed increased morbidity and mortality when treated with an anti-IFNAR antibody and 100% lethality in mice was reported between day 9 and 10 post-infection (VanBlargan et al., 2018). *Ifnar^−/−^* mice on the 129Sv/Ev background showed 100% lethality by day 5 following infection with WNV (Samuel and Diamond, 2005). Unlike these studies that have shown mortality by flaviviruses around day 5–10, we show that POWV LB strain infection resulted in 100% mortality rate in *Ifnar^−/−^* mice on the C57BL/6 background as early as 3 days after infection.

While no lethality was observed in *Ifnar^−/−^* mice (129Sv/Ev background) infected with Dengue virus (DENV2), virus was detected in the sera and organs harvested from infected animals (Shresta et al., 2004). Consistent with these results, we showed that *Ifnar^−/−^* mice are unable to control early viral burden. *Ifnar^−/−^* mice had significantly increased viremia compared to WT mice. It was also reported that IFN-α/β receptor plays an important role in resisting WNV by limiting tissue tropism (Daffis et al., 2009; Samuel and Diamond, 2005). Consistently, we showed that the lack of type-I IFN response led to significant viral dissemination in the peripheral tissues. *Ifnar^−/−^* mice had altered tissue tropism with significantly increased viral burden in the spleen, the liver and the kidney compared to WT mice. Previous studies have reported spleen and lymph nodes infection by POWV (Santos et al., 2016). Similarly, we showed spleen infection in both WT and *Ifnar^−/−^* mice following POWV infection. However, unlike WT mice, *Ifnar^−/−^* mice had significant histopathology and altered morphology in the spleen early after infection. Further, *Ifnar^−/−^* mice showed histopathology in the liver including hepatocyte swelling and infiltration of inflammatory cells while no significant pathology was detected in the WT mice.

Pro-inflammatory response is beneficial in recruiting immune cells to eliminate infectious viral factors. However, excessive inflammation can cause detrimental tissue damage (Pan et al., 2022). Type-I interferon response has a crucial role in regulating inflammation and its impairment can lead to the progression of inflammatory diseases (Ji et al., 2023). Additionally, impaired interferon response is associated with an exacerbated inflammatory response in severe and critical COVID-19 patients (Hadjadj et al., 2020). Consistently, following POWV infection, *Ifnar^−/−^* mice had increased levels of systemic inflammation. For example, we detected significant upregulation of several proinflammatory cytokines and chemokines including IL-6, CCL2, and CXCL10 in the sera of *Ifnar^−/−^* mice compared WT mice. Consistent with the increased viral load in *Ifnar^−/−^* mice, we also observed exacerbated inflammatory response in the spleen, liver and kidney.

Balanced immune response is required for protection against viral infections. It was recently reported that balanced T and B cell responses are necessary for immune protection against POWV infection (Stone et al., 2022). However, type-I interferon deficiency causes altered cellular tropism, with increased infection in splenic B cells, macrophages, and T cells during WNV infection (Samuel and Diamond, 2005). Similarly, we showed the increased infection of spleen in *Ifnar^−/−^* mice accompanied by a significant reduction in B cell numbers, monocytes, and neutrophils. Ly6C^+^ inflammatory monocytes (iMOs) are rapidly recruited to sites of infection and are continuously recruited into the spleen during infection (Terrazas et al., 2017). Ly6C^+^ monocytes are recruited to the brain and have a role in viral clearance in mice infected with WNV (Lim et al., 2011). Similarly, following POWV infection, we revealed a dramatic increase in the number of Ly6C^+^ cells in the spleen of WT mice. In contrast, we detected an impaired recruitment of Ly6C^+^ cells in POWV-infected *Ifnar^−/−^* spleens. Our observations are consistent with previous reports showing that under chronic inflammatory conditions, recruitment of Ly6C^+^ iMOs was abolished in IFNα/*β* receptor deficient mice (Lee et al., 2009).

It was previously shown that IFNα/β receptor deficient mice display uncontrolled WNV replication in the central nervous system (CNS) tissues (Samuel and Diamond, 2005). Herein, we did not detect any significant difference in viral burden in the brain of *Ifnar^−/−^* mice and WT mice at the time point examined. However, compared to WT mice, *Ifnar^−/−^* mice showed increased GFAP expression. The increased expression of GFAP suggests early glial activation following damage within the CNS (Pekny and Pekna, 2016; Potokar et al., 2019). *Ifnar^−/−^* mice also showed neuroinflammation marked by increased levels of proinflammatory cytokines and chemokines in the brain. For example, *Ifnar^−/−^* brains had significantly higher levels of IL-6. It was previously shown that WNV infection induces robust IL-6 production in mouse brains and several primary mouse cells (Kumar et al., 2010; Natekar et al., 2019). Notably, both WT and *Ifnar^−/−^* brains had significantly increased levels of CXCL10. Our results are consistent with previous reports showing that high CXCL10 expression is induced in human brain pericytes following tick-borne encephalitis virus infection (Prančlová et al., 2024).

In summary, this study is the first to demonstrate that type I interferon signaling plays a critical role in limiting POWV dissemination to peripheral organs and in modulating the inflammatory response. Mice deficient in IFNα/β receptor were highly susceptible to POWV infection. IFNα/β receptor deficiency resulted in uncontrolled viral burden and acute inflammation during POWV infection.

## Data Availability

The original contributions presented in the study are included in the article/supplementary material, further inquiries can be directed to the corresponding author.

## References

[ref1] Auroni T. T. Arora K. Natekar J. P. Pathak H. Elsharkawy A. Kumar M. (2023). The critical role of interleukin-6 in protection against neurotropic flavivirus infection. Front. Cell. Infect. Microbiol. 13:1275823. doi: 10.3389/fcimb.2023.1275823, PMID: 38053527 PMC10694511

[ref2] Basu M. Zurla C. Auroni T. T. Vanover D. Chaves L. C. S. Sadhwani H.. (2024). mRNA-encoded Cas13 can be used to treat dengue infections in mice. Nat. Microbiol. 9, 2160–2172. doi: 10.1038/s41564-024-01726-6, PMID: 38839984

[ref3] Browne A. S. Fang J. Elsharkawy A. Jia T. Reboli E. Luo Y.. (2025). Multilayer fluorescent immunoassay for early and sensitive dengue virus detection using host and viral biomarkers. Bioconjug. Chem. 36, 1474–1482. doi: 10.1021/acs.bioconjchem.5c00153, PMID: 40503688 PMC12272551

[ref4] Campbell O. Krause P. J. (2020). The emergence of human Powassan virus infection in North America. Ticks Tick-Borne Dis. 11:101540. doi: 10.1016/j.ttbdis.2020.101540, PMID: 32993949

[ref5] Chotiwan N. Rosendal E. Willekens S. M. A. Schexnaydre E. Nilsson E. Lindqvist R.. (2023). Type I interferon shapes brain distribution and tropism of tick-borne flavivirus. Nat. Commun. 14:2007. doi: 10.1038/s41467-023-37698-0, PMID: 37037810 PMC10086010

[ref6] Daffis S. Suthar M. S. Gale M. Diamond M. S. (2009). Measure and countermeasure: type I IFN (IFN-alpha/beta) antiviral response against West Nile virus. J. Innate Immun. 1, 435–445. doi: 10.1159/000226248, PMID: 20375601 PMC2920482

[ref7] Elsharkawy A. Dim C. Ge C. Patterson L. D. Nabi Z. Kumar M. (2025a). SARS-CoV-2 XBB.1.5 infects wild-type C57BL/6 mice and induces a protective CD4+ T cell response required for viral clearance. Front. Cell. Infect. Microbiol. 15:1621226. doi: 10.3389/fcimb.2025.1621226, PMID: 40822581 PMC12353745

[ref8] Elsharkawy A. Jahantigh H. R. Guglani A. Stone S. Arora K. Kumar M. (2025b). Virus-specific host responses and gene signatures following infection with major SARS-CoV-2 variants of concern: role of ZBP1 in viral clearance and lung inflammation. Front. Immunol. 16:1557535. doi: 10.3389/fimmu.2025.1557535, PMID: 40416961 PMC12098559

[ref9] Elsharkawy A. Stone S. Guglani A. Patterson L. D. Ge C. Dim C.. (2024). Omicron XBB.1.5 subvariant causes severe pulmonary disease in K18-hACE-2 mice. Front. Microbiol. 15:1466980. doi: 10.3389/fmicb.2024.1466980, PMID: 39417078 PMC11480052

[ref10] Fatmi S. S. Zehra R. Carpenter D. O. (2017). Powassan virus-a new reemerging tick-borne disease. Front. Public Health 5:342. doi: 10.3389/fpubh.2017.00342, PMID: 29312918 PMC5732952

[ref11] García-Sastre A. (2017). Ten strategies of interferon evasion by viruses. Cell Host Microbe 22, 176–184. doi: 10.1016/j.chom.2017.07.012, PMID: 28799903 PMC5576560

[ref12] Ge C. Salem A. R. Elsharkawy A. Natekar J. Guglani A. Doja J.. (2025). Development and characterization of a fully humanized ACE2 mouse model. BMC Biol. 23:194. doi: 10.1186/s12915-025-02293-w, PMID: 40597093 PMC12220410

[ref13] Gobillot T. A. Humes D. Sharma A. Kikawa C. Overbaugh J. (2020). The robust restriction of Zika virus by type-I interferon in A549 cells varies by viral lineage and is not determined by IFITM3. Viruses 12:503. doi: 10.3390/v12050503, PMID: 32370187 PMC7290589

[ref14] Hadjadj J. Yatim N. Barnabei L. Corneau A. Boussier J. Smith N.. (2020). Impaired type I interferon activity and inflammatory responses in severe COVID-19 patients. Science 369, 718–724. doi: 10.1126/science.abc6027, PMID: 32661059 PMC7402632

[ref15] Hassett E. M. Thangamani S. (2021). Ecology of Powassan virus in the United States. Microorganisms 9:2317. doi: 10.3390/microorganisms9112317, PMID: 34835443 PMC8624383

[ref16] Hermance M. E. Thangamani S. (2017). Powassan virus: an emerging arbovirus of public health concern in North America. Vector Borne Zoonotic Dis. 17, 453–462. doi: 10.1089/vbz.2017.2110, PMID: 28498740 PMC5512300

[ref17] Jahantigh H. R. Elsharkawy A. Guglani A. Arora K. Patterson L. D. Kumar M. (2025). Neurobiological alterations induced by SARS-CoV-2: insights from variant-specific host gene expression patterns in hACE2-expressing mice. Viruses 17:329. doi: 10.3390/v17030329, PMID: 40143258 PMC11946589

[ref18] Ji L. Li T. Chen H. Yang Y. Lu E. Liu J.. (2023). The crucial regulatory role of type I interferon in inflammatory diseases. Cell Biosci. 13:230. doi: 10.1186/s13578-023-01188-z, PMID: 38124132 PMC10734085

[ref19] Keller B. C. Fredericksen B. L. Samuel M. A. Mock R. E. Mason P. W. Diamond M. S.. (2006). Resistance to alpha/beta interferon is a determinant of West Nile virus replication fitness and virulence. J. Virol. 80, 9424–9434. doi: 10.1128/JVI.00768-06, PMID: 16973548 PMC1617238

[ref20] Kumar M. Roe K. O’Connell M. Nerurkar V. R. (2015). Induction of virus-specific effector immune cell response limits virus replication and severe disease in mice infected with non-lethal West Nile virus Eg101 strain. J. Neuroinflammation 12:178. doi: 10.1186/s12974-015-0400-y, PMID: 26392176 PMC4578235

[ref21] Kumar M. Verma S. Nerurkar V. R. (2010). Pro-inflammatory cytokines derived from West Nile virus (WNV)-infected SK-N-SH cells mediate neuroinflammatory markers and neuronal death. J. Neuroinflammation 7:73. doi: 10.1186/1742-2094-7-73, PMID: 21034511 PMC2984415

[ref22] Lazear H. M. Govero J. Smith A. M. Platt D. J. Fernandez E. Miner J. J.. (2016). A mouse model of Zika virus pathogenesis. Cell Host Microbe 19, 720–730. doi: 10.1016/j.chom.2016.03.010, PMID: 27066744 PMC4866885

[ref23] Lee P. Y. Li Y. Kumagai Y. Xu Y. Weinstein J. S. Kellner E. S.. (2009). Type I interferon modulates monocyte recruitment and maturation in chronic inflammation. Am. J. Pathol. 175, 2023–2033. doi: 10.2353/ajpath.2009.090328, PMID: 19808647 PMC2774066

[ref24] Li X.-F. Li X.-D. Deng C.-L. Dong H.-L. Zhang Q.-Y. Ye Q.. (2017). Visualization of a neurotropic flavivirus infection in mouse reveals unique viscerotropism controlled by host type I interferon signaling. Theranostics 7, 912–925. doi: 10.7150/thno.16615, PMID: 28382163 PMC5381253

[ref25] Lim J. K. Obara C. J. Rivollier A. Pletnev A. G. Kelsall B. L. Murphy P. M. (2011). Chemokine receptor Ccr2 is critical for monocyte accumulation and survival in West Nile virus encephalitis. J. Immunol. 186, 471–478. doi: 10.4049/jimmunol.1003003, PMID: 21131425 PMC3402345

[ref26] Lubick K. J. Robertson S. J. McNally K. L. Freedman B. A. Rasmussen A. L. Taylor R. T.. (2015). Flavivirus antagonism of type i interferon signaling reveals prolidase as a regulator of IFNAR1 surface expression. Cell Host Microbe 18, 61–74. doi: 10.1016/j.chom.2015.06.007 26159719 PMC4505794

[ref27] Mazeaud C. Freppel W. Chatel-Chaix L. (2018). The multiples fates of the Flavivirus RNA genome during pathogenesis. Front. Genet. 9:595. doi: 10.3389/fgene.2018.00595, PMID: 30564270 PMC6288177

[ref28] McLEAN D. M. Donohue W. L. (1959). Powassan virus: isolation of virus from a fatal case of encephalitis. Can. Med. Assoc. J. 80, 708–711. 13652010 PMC1830849

[ref29] McNab F. Mayer-Barber K. Sher A. Wack A. O’Garra A. (2015). Type i interferons in infectious disease. Nat. Rev. Immunol. 15, 87–103. doi: 10.1038/nri3787 25614319 PMC7162685

[ref30] Natekar J. P. Rothan H. A. Arora K. Strate P. G. Kumar M. (2019). Cellular microrna-155 regulates virus-induced inflammatory response and protects against lethal West Nile virus infection. Viruses 12:9. doi: 10.3390/v12010009, PMID: 31861621 PMC7019255

[ref31] O’Brien C. A. Hobson-Peters J. Yam A. W. Y. Colmant A. M. G. McLean B. J. Prow N. A.. (2015). Viral RNA intermediates as targets for detection and discovery of novel and emerging mosquito-borne viruses. PLoS Negl. Trop. Dis. 9:e0003629. doi: 10.1371/journal.pntd.0003629, PMID: 25799391 PMC4370754

[ref32] O’Neill L. A. J. Bowie A. G. (2010). Sensing and signaling in antiviral innate immunity. Curr. Biol. 20, R328–R333. doi: 10.1016/j.cub.2010.01.044 20392426

[ref33] Oh S.-J. Kumari P. Auroni T. T. Stone S. Pathak H. Elsharkawy A.. (2024). Upregulation of neuroinflammation-associated genes in the brain of SARS-CoV-2-infected mice. Pathogens 13:528. doi: 10.3390/pathogens13070528, PMID: 39057755 PMC11280415

[ref34] Pan Y. Cai W. Cheng A. Wang M. Yin Z. Jia R. (2022). Flaviviruses: innate immunity, Inflammasome activation, inflammatory cell death, and cytokines. Front. Immunol. 13:829433. doi: 10.3389/fimmu.2022.829433, PMID: 35154151 PMC8835115

[ref35] Pekny M. Pekna M. (2016). Reactive gliosis in the pathogenesis of CNS diseases. Biochim. Biophys. Acta 1862, 483–491. doi: 10.1016/j.bbadis.2015.11.014, PMID: 26655603

[ref36] Potokar M. Jorgačevski J. Zorec R. (2019). Astrocytes in Flavivirus infections. Int. J. Mol. Sci. 20:691. doi: 10.3390/ijms20030691, PMID: 30736273 PMC6386967

[ref37] Prančlová V. Hönig V. Zemanová M. Růžek D. Palus M. (2024). Robust CXCL10/IP-10 and CCL5/RANTES production induced by tick-borne encephalitis virus in human brain Pericytes despite weak infection. Int. J. Mol. Sci. 25:7892. doi: 10.3390/ijms25147892, PMID: 39063134 PMC11276942

[ref38] Samuel M. A. Diamond M. S. (2005). Alpha/beta interferon protects against lethal West Nile virus infection by restricting cellular tropism and enhancing neuronal survival. J. Virol. 79, 13350–13361. doi: 10.1128/JVI.79.21.13350-13361.2005, PMID: 16227257 PMC1262587

[ref39] Santos R. I. Hermance M. E. Gelman B. B. Thangamani S. (2016). Spinal cord ventral horns and lymphoid organ involvement in Powassan virus infection in a mouse model. Viruses 8:220. doi: 10.3390/v8080220, PMID: 27529273 PMC4997582

[ref40] Shresta S. Kyle J. L. Snider H. M. Basavapatna M. Beatty P. R. Harris E. (2004). Interferon-dependent immunity is essential for resistance to primary dengue virus infection in mice, whereas T- and B-cell-dependent immunity are less critical. J. Virol. 78, 2701–2710. doi: 10.1128/jvi.78.6.2701-2710.2004, PMID: 14990690 PMC353772

[ref41] Son K.-N. Liang Z. Lipton H. L. (2015). Double-stranded RNA is detected by immunofluorescence analysis in RNA and DNA virus infections, including those by negative-stranded RNA viruses. J. Virol. 89, 9383–9392. doi: 10.1128/JVI.01299-15, PMID: 26136565 PMC4542381

[ref42] Stone S. Elsharkawy A. Burleson J. D. Hauser M. Domi A. Kumari P.. (2025). Multi-antigen viral-vectored vaccine protects against SARS-CoV-2 and variants in a lethal hACE2 transgenic mouse model. Vaccine 13:411. doi: 10.3390/vaccines13040411, PMID: 40333327 PMC12031414

[ref43] Stone E. T. Hassert M. Geerling E. Wagner C. Brien J. D. Ebel G. D.. (2022). Balanced T and B cell responses are required for immune protection against Powassan virus in virus-like particle vaccination. Cell Rep. 38:110388. doi: 10.1016/j.celrep.2022.110388, PMID: 35172138 PMC8919300

[ref44] Terrazas C. Varikuti S. Oghumu S. Steinkamp H. M. Ardic N. Kimble J.. (2017). Ly6Chi inflammatory monocytes promote susceptibility to Leishmania donovani infection. Sci. Rep. 7:14693. doi: 10.1038/s41598-017-14935-3, PMID: 29089636 PMC5665970

[ref45] Trinchieri G. (2010). Type I interferon: friend or foe? J. Exp. Med. 207, 2053–2063. doi: 10.1084/jem.20101664, PMID: 20837696 PMC2947062

[ref46] VanBlargan L. A. Himansu S. Foreman B. M. Ebel G. D. Pierson T. C. Diamond M. S. (2018). An mRNA vaccine protects mice against multiple tick-transmitted Flavivirus infections. Cell Rep. 25, 3382–3392.e3. doi: 10.1016/j.celrep.2018.11.082, PMID: 30566864 PMC6353567

[ref47] Weber E. Finsterbusch K. Lindquist R. Nair S. Lienenklaus S. Gekara N. O.. (2014). Type I interferon protects mice from fatal neurotropic infection with Langat virus by systemic and local antiviral responses. J. Virol. 88, 12202–12212. doi: 10.1128/JVI.01215-14, PMID: 25122777 PMC4248936

[ref48] Wei J. Ma Y. Wang L. Chi X. Yan R. Wang S.. (2017). Alpha/beta interferon receptor deficiency in mice significantly enhances susceptibility of the animals to pseudorabies virus infection. Vet. Microbiol. 203, 234–244. doi: 10.1016/j.vetmic.2017.03.022, PMID: 28619150

